# An improvement of current hypercube pooling PCR tests for SARS-CoV-2 detection

**DOI:** 10.3389/fpubh.2022.994712

**Published:** 2022-10-19

**Authors:** Tai-Yin Wu, Yu-Ciao Liao, Chiou-Shann Fuh, Pei-Wei Weng, Jr-Yi Wang, Chih-Yu Chen, Yu-Min Huang, Chung-Pei Chen, Yo-Lun Chu, Cheng-Kuang Chen, Kuei-Lin Yeh, Ching-Hsiao Yu, Hung-Kang Wu, Wei-Peng Lin, Tsan-Hon Liou, Mai-Szu Wu, Chen-Kun Liaw

**Affiliations:** ^1^Department of Family Medicine, Zhongxing Branch, Taipei City Hospital, Taipei, Taiwan; ^2^Institute of Epidemiology and Preventive Medicine, National Taiwan University, Taipei, Taiwan; ^3^General Education Center, University of Taipei, Taipei, Taiwan; ^4^Institute of Computer Science and Information Engineering, National Taiwan University, Taipei, Taiwan; ^5^Department of Orthopedics, School of Medicine, College of Medicine, Taipei Medical University, Taipei, Taiwan; ^6^Department of Orthopedics, Shuang Ho Hospital, Taipei Medical University, New Taipei, Taiwan; ^7^Graduate Institute of Biomedical Optomechatronics, College of Biomedical Engineering, Research Center of Biomedical Device, Taipei Medical University, Taipei, Taiwan; ^8^International Ph.D. Program in Biomedical Engineering, College of Biomedical Engineering, Taipei Medical University, Taipei, Taiwan; ^9^Department of Orthopedics, Cathay General Hospital, Taipei, Taiwan; ^10^Department of Orthopedics, Shin Kong Wu Ho-Su Memorial Hospital, Taipei, Taiwan; ^11^School of Medicine, College of Medicine, Fu Jen Catholic University, Taipei, Taiwan; ^12^Department of Biomedical Engineering, National Taiwan University, Taipei, Taiwan; ^13^Department of Orthopaedics, Ditmanson Medical Foundation Chia-Yi Christian Hospital, Chiayi, Taiwan; ^14^Department of Long-Term Care and Management, WuFeng University, Chiayi, Taiwan; ^15^Department of Orthopaedic Surgery, Taoyuan General Hospital, Ministry of Health and Welfare, Taoyuan, Taiwan; ^16^Department of Orthopaedic Surgery, National Taiwan University Hospital, Taipei, Taiwan; ^17^Department of Nursing, Yuanpei University of Medical Technology, Hsinchu, Taiwan; ^18^Department of Orthopedics, Postal Hospital, Taipei, Taiwan; ^19^Department of Physical Medicine and Rehabilitation, School of Medicine, College of Medicine, Taipei Medical University, Taipei, Taiwan; ^20^Division of Nephrology, School of Medicine, College of Medicine, Taipei Medical University, Taipei, Taiwan; ^21^TMU Biodesign Center, Taipei Medical University, Taipei, Taiwan

**Keywords:** pooling PCR, hypercube pooling, SARS-CoV-2, COVID-19, one round pooling PCR

## Abstract

The severe acute respiratory syndrome coronavirus 2 (SARS-CoV-2) pandemic can be effectively controlled by rapid and accurate identification of SARS-CoV-2-infected cases through large-scale screening. Hypercube pooling polymerase chain reaction (PCR) is frequently used as a pooling technique because of its high speed and efficiency. We attempted to implement the hypercube pooling strategy and found it had a large quantization effect. This raised two questions: is hypercube pooling with edge = 3 actually the optimal strategy? If not, what is the best edge and dimension? We used a C++ program to calculate the expected number of PCR tests per patient for different values of prevalence, edge, and dimension. The results showed that every edge had a best performance range. Then, using C++ again, we created a program to calculate the optimal edge and dimension required for pooling samples when entering prevalence into our program. Our program will be provided as freeware in the hope that it can help governments fight the SARS-CoV-2 pandemic.

## Introduction

The severe acute respiratory syndrome coronavirus 2 (SARS-CoV-2) pandemic has drastically changed the life of people worldwide. Currently, there are two strategies that are being used to contain the spread of the virus. One method is to impose strict restrictions on normal activities, such as China and many other countries imposing severe lockdowns. The other is to achieve high vaccination coverage. For example, the vaccination coverages in the United Kingdom and Unites States are both relatively high.

Effective control of SARS-CoV-2 infections requires rapid and precise identification of infected people through large-scale polymerase chain reaction (PCR) tests. However, no country has enough time and resources to test each individual sample separately. One efficient alternative, which many countries have adopted, is to pool samples and test them together. Many pooling strategies and techniques have been proposed (1–35).

**Figure 1:**
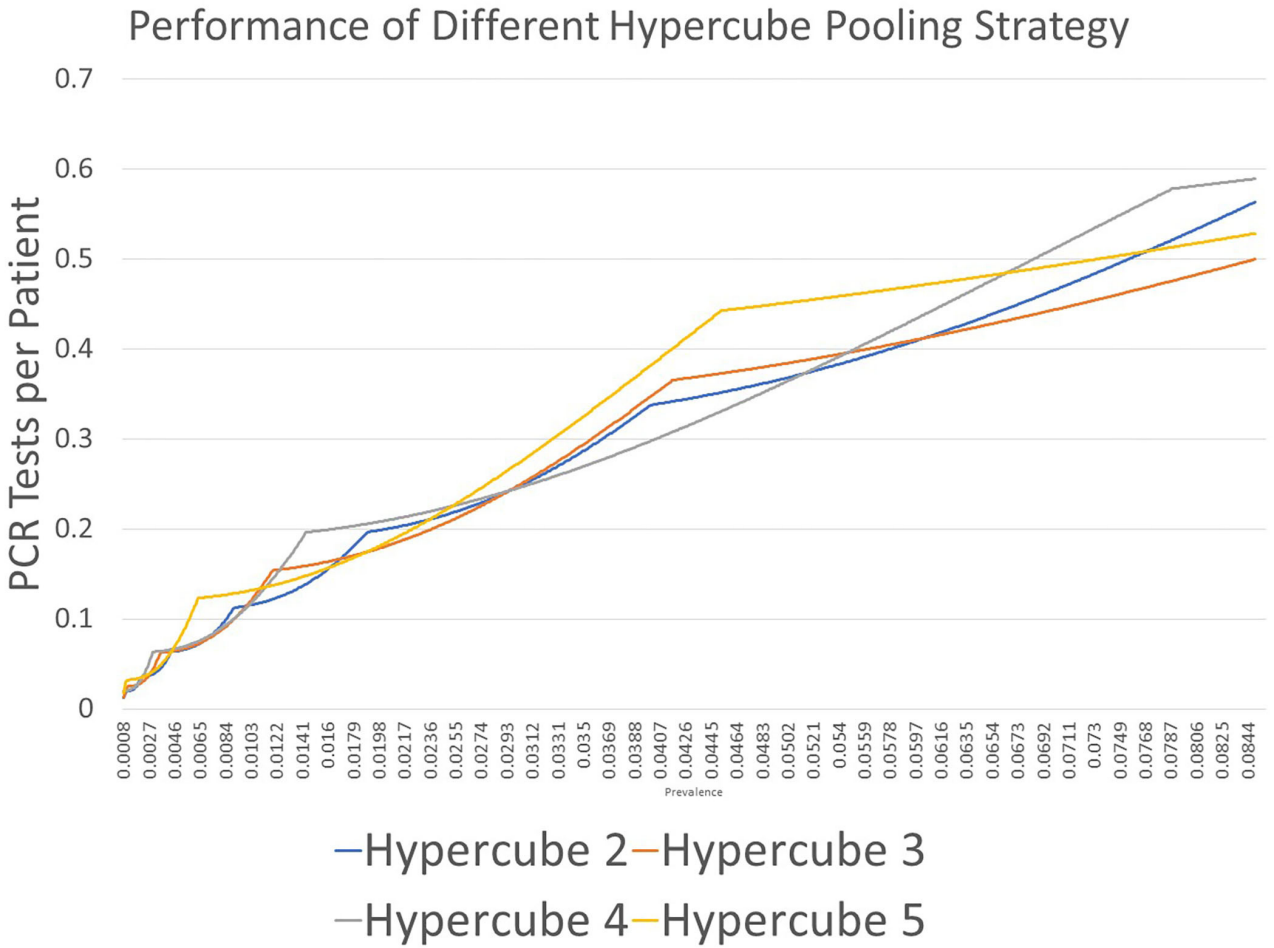
Results of PCR tests for each patient using the hypercube pooling strategy with edge values of 2 (Hypercube 2), 3 (Hypercube 3), 4 (Hypercube 4), and 5 (Hypercube 5). The quantization effects are displayed. Every strategy has an area in which its performance is strong.

Mutesa et al. proposed hypercube pooling, which is both efficient and fast (36, 37). The authors proposed edge three to be the most suitable edge, and the dimension can be calculated by ln (0.35/p) where p is the prevalence. Then, pooling is illustrated in Figure 1.

We observed a significant quantization effect during the use of hypercube pooling, which dramatically increases the efficiency despite a minor prevalence change. This raised our interest to explore the underlying mechanism of this method. Mutesa et al. (36, 37) suggested that using edge three in hypercubic pooling should be the most efficient strategy. In that case, we wondered the following: (1) if the quantization effect does influence efficiency, is hypercube pooling with edge three really the best strategy? And (2), if the answer is no, what is the best edge and dimension?

## Methods

The approach to selecting the estimated number of PCR tests per patient was first constructed in accordance with the concept of the hypercubic method and its variables: prevalence, edge, and dimension.

By using probability mathematics, the formula for the estimated number of PCR tests per patient was established as follows:


$$\begin{array}{l} =\text{ total number of tests}/\text{total number of patients}\\ =\;(\text{total number of scan tests }+\text{ estimated number of}\\ \text{ additional tests})/{\text{edge}}^{\text{ dimension}}\\ =(\text{total number of scan tests}\\ \;+\;{\left(\text{estimated frequency of positive scans}\right)}^{\text{dimension}})/n^{d} \end{array}$$

(1) $$\begin{array}{l} =\frac{d\;*\;n+{\left(n(1-{\left(1-p\right)}^{\left(n^{\left(d-1\right)}\right)})\right)}^{d}}{n^{d}}\\ =\frac{d\;*\;n}{n^{d}}+\frac{{\left(n(1-{\left(1-p\right)}^{\left(n^{\left(d-1\right)}\right)})\right)}^{d}}{n^{d}}\\ =\frac{d}{n^{\left(d-1\right)}}+{\left(1-{\left(1-p\right)}^{\left(n^{\left(d-1\right)}\right)}\right)}^{d} \end{array}$$

where *n* is the edge, *d* is the dimension, and *p* is the prevalence.

We used *n* = 2, 3, 4, and 5 in Formula (1) to determine which strategy works best under various values for the disease prevalence *p*.

C++ was used for implementation.

By leveraging a computer's computational ability, we simply computed every test possibility by using the brute force method. We then assigned dimension = 2 and edge = 2 in the formula to yield the maximal edge and dimension, respectively. Subsequently, we used the formula to calculate the estimates from edge = 2 to the maximal edge and from dimension = 2 to the maximal dimension, and then we chose the smallest number from all the estimated numbers of PCR tests per patient (1).

## Results

**Figure 2:**
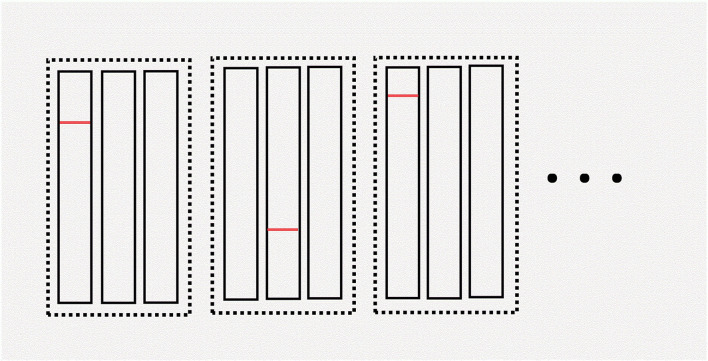
Illustration of hypercube pooling. The “dimension” is the total number of dotted rectangles. The “edge” is the number of rectangles within each dotted rectangle. The red line in this figure denotes a positive sample. In this example, the first pool in the first group, the second pool in the second group, and the first pool in the third group are all positive. We can determine which patient is positive during this round of pooling by using the hypercube pooling strategy. For more details about hypercube pooling, the readers can refer to previous literature (36, 37).

Figure 2 shows the result of the first part of the study. We demonstrated that every proposed strategy had an area in which its performance was best. To obtain the best edge and dimension for the highest performance under a specific prevalence, we needed to conduct the second part of the investigation. For example, using edge = 2 (Hypercube 2 in Figure 2) resulted in the lowest estimate for the number of PCR tests per patient at prevalence of 0.0123–0.0537; at prevalence between 0.0261 and 0.0744, edge = 3 (Hypercube 3 in Figure 2) resulted in the lowest estimate.

**Figure 3:**
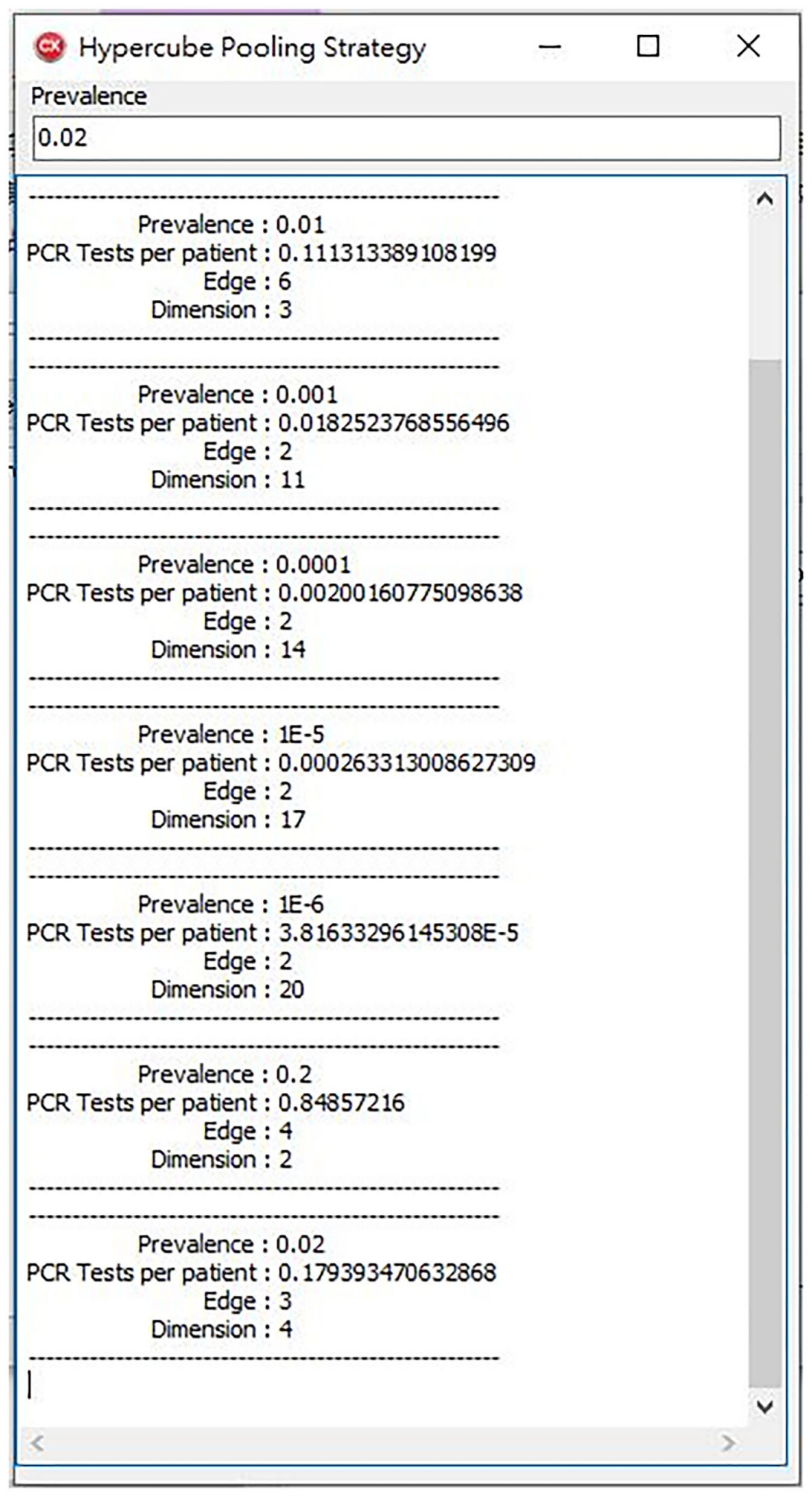
Our program offers a high-performance hypercube pooling strategy. The dimension, edge, and expected number of PCR tests per patient are the output variables.

Therefore, we wrote a program for the second part of the study. The program uses the brute force method that examines every possible dimension number and edge number. Then, we calculated its performance by Formula (1). Finally, we chose the one which performs the best. When we entered the prevalence, the program calculated the best performance edge, dimension, and estimated number of PCR tests per patient (Figure 3).

## Discussion

Various pooling techniques have been described, such as single pooling, n n double pooling, n^2^ n double pooling, and array pooling. In our opinion, one-dimensional hypercube pooling is single pooling, and array pooling is two-dimensional pooling.

Some pooling strategies—such as no pooling, array pooling, and hypercube pooling—can identify infected patients with a single round of PCR tests. According to a study by Mutesa et al., we can be 96% certain of positive cases in just the first round of pooled PCR tests (36, 37). Only 4% require second-round PCR tests. Hypercube pooling is a fast and efficient pooling method. Furthermore, a similar pooling approach was proposed almost at the same time (1). However, the authors did not explicitly mention the method or how it calculated the optimal pooling size. In this study, we made the complex probability mathematics more calculable by switching it and writing it into a program. Our method is much simpler and clearer than other methods.

Every PCR machine that uses a specific primer has a different maximal allowable dilution. However, the cutoff cycle threshold (Ct) can also change the maximum dilution. Therefore, we should conduct a pilot study to understand the limits of our machine. Additionally, we cannot pool more samples during hypercube pooling than the maximal allowable dilution.


(2) $$\begin{array}{l} \text{That is}:\text{ maximal allowable dilution }>\text{ edg}{\text{e}}^{\left(\text{dimension }-\;1\right)} \end{array}$$

Another challenge in the use of this method is the high number of dimensions. Two dimensions or even three dimensions are easy to imagine and visualize, but, in higher dimensions, we may need to code every sample with a specific number to identify individuals with infection accurately and continue the hypercube pooling process.

The prevalence of infection is unknown when conducting large-scale screening. Therefore, we recommend to begin pooling with edge = 2, which produces the maximum dimension that satisfies Equation (2). Subsequently, the prevalence can be calculated, and the strategy for the rest of the tests can be adjusted.

However, pooling may not yield significant benefits in areas with high disease prevalence. If a prevalence value is entered into the software and the expected number of PCR tests per patient is greater than one, pooling is not necessary. Instead, pooling will waste time and resources.

## Conclusion

This pooling approach for SARS-CoV-2 detection is hypercube pooling PCR with an optimal edge and a dimension. This type of pooling can be completed in only one round of testing. We offer a tool to calculate the edge and the dimension required for pooling. We hope that it can be widely used, especially in large-scale screening, to better detect SARS-CoV-2 cases.

## Data availability statement

The original contributions presented in the study are included in the article/supplementary material, further inquiries can be directed to the corresponding author/s.

## Author contributions

CK-L, T-YW, and Y-CL conceptualized the study and wrote the paper. CK-L, P-WW, J-YW, C-YC, and Y-MH helped acquire the funding. Y-CL deduced the mathematics. C-SF, C-PC, Y-LC, C-KC, and K-LY wrote an algorithm. C-HY, H-KW, W-PL, T-HL, M-SW, and Y-MH simulated data and tested. All authors edited the paper. All authors contributed to the article and approved the submitted version.

## Funding

This work was funded by grants from the Taiwan Ministry of Science and Technology (Grant No. 109-2314-B-038-029).

## Conflict of interest

The authors declare that the research was conducted in the absence of any commercial or financial relationships that could be construed as a potential conflict of interest.

## Publisher's note

All claims expressed in this article are solely those of the authors and do not necessarily represent those of their affiliated organizations, or those of the publisher, the editors and the reviewers. Any product that may be evaluated in this article, or claim that may be made by its manufacturer, is not guaranteed or endorsed by the publisher.
